# Joint disease mapping using six cancers in the Yorkshire region of England

**DOI:** 10.1186/1476-072X-7-41

**Published:** 2008-07-28

**Authors:** Amy Downing, David Forman, Mark S Gilthorpe, Kimberley L Edwards, Samuel OM Manda

**Affiliations:** 1 Cancer Epidemiology Group, Centre for Epidemiology & Biostatistics, University of Leeds, Room 8.49 Worsley Building, Leeds, LS2 9LN, UK; 2 Northern & Yorkshire Cancer Registry & Information Service, Bexley Wing, St James's University Hospital, Beckett Street, Leeds, LS9 7TF, UK; 3 Biostatistics Unit, Centre for Epidemiology & Biostatistics, University of Leeds, Room 8.49 Worsley Building, Leeds, LS2 9LN, UK; 4 Biostatistics Unit, South African Medical Research Council, 1 Soutpansberg Road, Pretoria, Private Bag x385, 0001, Pretoria, South Africa

## Abstract

**Objectives:**

The aims of this study were to model jointly the incidence rates of six smoking related cancers in the Yorkshire region of England, to explore the patterns of spatial correlation amongst them, and to estimate the relative weight of smoking and other shared risk factors for the relevant disease sites, both before and after adjustment for socioeconomic background (SEB).

**Methods:**

Data on the incidence of oesophagus, stomach, pancreas, lung, kidney, and bladder cancers between 1983 and 2003 were extracted from the Northern & Yorkshire Cancer Registry database for the 532 electoral wards in the Yorkshire region. Using postcode of residence, each case was assigned an area-based measure of SEB using the Townsend index. Standardised incidence ratios (SIRs) were calculated for each cancer site and their correlations investigated. The joint analysis of the spatial variation in incidence used a Bayesian shared-component model. Three components were included to represent differences in smoking (for all six sites), bodyweight/obesity (for oesophagus, pancreas and kidney cancers) and diet/alcohol consumption (for oesophagus and stomach cancers).

**Results:**

The incidence of cancers of the oesophagus, pancreas, kidney, and bladder was relatively evenly distributed across the region. The incidence of stomach and lung cancers was more clustered around the urban areas in the south of the region, and these two cancers were significantly associated with higher levels of area deprivation. The incidence of lung cancer was most impacted by adjustment for SEB, with the rural/urban split becoming less apparent. The component representing smoking had a larger effect on cancer incidence in the eastern part of the region. The effects of the other two components were small and disappeared after adjustment for SEB.

**Conclusion:**

This study demonstrates the feasibility of joint disease modelling using data from six cancer sites. Incidence estimates are more precise than those obtained without smoothing. This methodology may be an important tool to help authorities evaluate healthcare system performance and the impact of policies.

## Background

Mapping of the geographical distribution of cancer incidence, survival, or mortality rates can help us understand spatial patterns of disease and identify differences in disease burden across an area. These maps can be used by those involved in the planning of services or in cancer prevention and control programmes, both locally and nationally, and can provide useful background information for academics, the government, and the general public. For example, the Cancer E-Atlas provides basic statistics (available as maps) on the incidence of and mortality from the most common cancers across England [1]. Such maps tend to show relative risk (RR), expressed as population-based standardised incidence or mortality ratios (SIRs/SMRs) and can be calculated at varying levels of geographical resolution. There is a need to maintain a balance between preserving patient privacy and obtaining fine geographical resolution, and in the UK this is commonly overcome by using the unit of electoral wards, which include an average of 5,000 residents. However, population-based rates across such relatively small geographical areas can vary greatly due to the differences in population sizes and incidence rates. Furthermore, derived SIRs/SMRs can be unstable for areas with small populations[2]. One way to address this problem is to use spatial smoothing techniques that recognise that observations from the same area share similar properties. In this way the variability or 'noise' can be reduced. Many different approaches to spatial smoothing have been developed, but the one that has gained wide acceptance and applicability is that of Besag, York and Mollie (the BYM model), which allows for both heterogeneous and spatially structured random effects[3].

Much of the work in the area of spatial smoothing has focused on the modelling of a single disease. However, many diseases share common risk factors and more recently joint disease mapping has emerged. By 'borrowing' information from different diseases, the estimates (rates) can be further improved [4]. Several methods for joint disease mapping have been proposed, using both Bayesian and non-Bayesian techniques [5-9]. Manda & Leyland compared maximum likelihood (frequentist) and Bayesian estimation methods using Markov Chain Monte Carlo (MCMC) for multivariate spatial disease models[10]. They found that whilst the parameters are estimated similarly under the two methods, random effects variance estimates are generally attenuated under the frequentist approach compared to the Bayesian approach. They suggest that MCMC is easier to implement and provides more reliable estimates for many realistic epidemiological problems. Assunção and Castro showed that disease incidence rates obtained using a multivariate Bayesian model were more precise, i.e. with smaller confidence intervals (CIs), compared to those obtained with classical indirect standardisation, which had very large CIs due to the small number of cases. However, this analysis did not consider spatial effects[4].

A further extension is the inclusion of shared components[8], which are common factors shared by different subsets of diseases. The area-specific values of these shared components and the relative contribution (weight) of the component to each relevant disease may be estimated[11]. One of the most common shared risk factors for cancer is cigarette smoking[12]. In this study we chose to model the incidence rates of six cancers for which smoking is a major risk factor; cancers of the oesophagus, stomach, pancreas, lung, kidney, and bladder. These are amongst the 15 most common cancers in the Yorkshire region of England and together account for approximately 30% of all cancer cases[13]. In addition to smoking, these cancers share other risk factors; oesophagus, pancreas, and kidney cancers are linked to bodyweight/obesity; oesophagus and stomach cancer are associated with diet (including alcohol consumption)[12]. The aims of this study were to model jointly the incidence rates of these six cancers in the Yorkshire region, to explore the patterns of spatial correlation amongst them, and to estimate the relative weight of the shared risk factors for each site, both before and after adjustment for socioeconomic background (SEB).

## Methods

The six cancer sites included in this study were oesophagus (ICD10 code C15), stomach (C16), pancreas (C25), lung (C33-34), kidney (C64), and bladder (C67). Data on cancer incidence between 1983 and 2003 were extracted from the Northern & Yorkshire Cancer Registry & Information Service database for the 532 electoral wards in the Yorkshire Health Region according to the 1991 census. Using the postcode of residence at the time of diagnosis, each person was assigned an area-based measure of SEB using the Townsend index [14]. SIRs were calculated for each cancer site (with the number of expected cases calculated using the average number of cases per ward observed in Yorkshire and the population in 1991) and correlations amongst these were investigated.

The methodology used was an alternative to that described in Langford et al[5], Leyland et al [6], Assunção and Castro [4], and Feltbower et al[15], whereby joint modelling of multiple diseases is achieved using a multivariate normal structure on relative risks, the first two using non-Bayesian methods and the later two using Bayesian formulations. In this study, the joint analysis of the spatial variation of incidence rates of the six cancers was modelled by the shared-component model, in which different cancers share latent spatial components. We formulated the joint modelling from a Bayesian perspective, similar to that described in Held et al[16], which is an extension of the formulations described initially in Knorr-Held and Best[8] for the two-disease setting. The common feature of this class of shared-common models is that the latent components act as surrogates for geographical variations of the unobserved spatially-structured risk factors that affect some or all diseases.

Suppose that the region under consideration has been divided into *I *contiguous sub-regions; these are areas or electoral wards for the Yorkshire region in our case. Let *O*_*ij *_represent the observed number of incident cases of cancer *j *in area *i *(1 ≤ *i *≤ *I *= 532, 1 ≤ *j *≤ 6, in our case). It is assumed that the observed counts follow Poisson distributions with mean *μ*_*ij *_= *E*_*ij*_*R*_*ij*_, where *E*_*ij *_and *R*_*ij *_are the known expected number of cases and unknown relative risk, respectively, in area *i *for disease *j*. The maximum likelihood estimate of the relative risk of disease *j *in area *i *is the usual standardised morbidity/incidence ratio ${\hat{R}}_{ij}$ = *O*_*ij*_/*E*_*ij*_. It is well known that these crude risk ratios are misleading, particularly when the diseases are rare or the areas are small. Thus, more reliable estimates of relative risks for rare diseases or small areas can be obtained by borrowing information from neighbouring areas.

The basic BYM (Besag, York and Mollie) model [3] decomposes the log of disease-specific area-level relative risks into the sum of two random effects: one which is unstructured (heterogeneous) and the other spatially structured (dependent). The unstructured random effects are assumed to follow a normal distribution. The spatially structured effects are modelled by the intrinsic conditional autoregressive normal (CAR Normal) prior, which, in simple formulation, specifies that the conditional distribution of each area-specific spatially structured effect, given all other spatial effects, is a normal distribution with mean equal to the average of its bordering neighbours, and variance inversely proportional to the number of these neighbours; the more neighbours an area has, the greater the precision is for that area effect.

Rather than using a multivariate normal prior to assess spatial correlations amongst the six cancers, we used a shared-component model [8,16]. In our model, we included three shared components (chosen *a priori *based on common risk factors[12]): the first relevant to all six cancer sites, which we interpret to act as a surrogate for variations in smoking; the second representing differences in bodyweight/obesity for oesophagus, pancreas, and kidney; the third for oesophagus and stomach cancer representing variations in diet/alcohol consumption. We also included the relative weights of each common component for the relevant cancers. The resulting model enables us to determine the extent of the variation exhibited through common geographical patterns in diseases in space. Thus, following Held et al, we modelled the log relative risk as:

$$\begin{array}{c} \log(R_{i1})=\alpha_{1}+\beta_{1}^{T}x_{i}+\varphi_{1i}\kappa_{1,1}+\varphi_{2i}\kappa_{2,1}+\varphi_{3i}\kappa_{3,1}+\varepsilon_{i1}\\ \log(R_{i2})=\alpha_{2}+\beta_{2}^{T}x_{i}+\varphi_{1i}\kappa_{1,2}+\varphi_{3i}\kappa_{3,2}+\varepsilon_{i2}\\ \log(R_{i3})=\alpha_{3}+\beta_{3}^{T}x_{i}+\varphi_{1i}\kappa_{1,3}+\varphi_{2i}\kappa_{2,2}+\varepsilon_{i3}\\ \log(R_{i4})=\alpha_{4}+\beta_{4}^{T}x_{i}+\varphi_{1i}\kappa_{1,4}+\varepsilon_{i4}\\ \log(R_{i5})=\alpha_{5}+\beta_{5}^{T}x_{i}+\varphi_{1i}\kappa_{1,5}+\varphi_{2i}\kappa_{2,3}+\varepsilon_{i5}\\ \log(R_{i6})=\alpha_{6}+\beta_{6}^{T}x_{i}+\varphi_{1i}\kappa_{1,6}+\varepsilon_{i6} \end{array}$$

where *R*_*i*1 _is the log relative risk for oesophagus cancer in ward *i*, *R*_*i*2 _is the log relative risk for stomach cancer, and likewise *R*_*i*3_, *R*_*i*4_, *R*_*i*5 _and *R*_*i*6 _for pancreas, lung, kidney, and bladder cancers, respectively. The parameter *α*_*j *_is the disease-specific intercept and *β*_*j *_are the disease-specific risk coefficients associated with the risk vector *x*; *φ*_1*i *_is the shared *smoking *component common to all six cancers; *φ*_2*i *_is the shared *bodyweight/obesity *component relevant to oesophagus, pancreas, and kidney cancers only; and *φ*_3*i *_is the shared *diet/alcohol *component for oesophagus and stomach cancers only. The unknown parameters *κ *allow for different risk gradients for the relevant diseases and *ε*_*ij *_are the disease-specific heterogeneous effects, capturing possible extra-Poisson variation that is not explained by the included terms.

For a Bayesian model to be completed, all unknown parameters, whether for fixed or random effects, are given prior distributions. Where prior knowledge is available, this should be reflected in the prior distributions, otherwise prior ignorance is assumed on the distributions of parameters. We want priors that combine the BYM framework to link risk in space. For the purpose of this application, the shared spatial random effects *φ*_*i *_were given a prior distribution to capture local dependence in space. The disease-specific heterogeneity terms were modelled to arise from a multivariate normal prior distribution with covariance matrix Σ to allow for correlations amongst the cancers. Since we used the CAR Normal prior, with sum-to-zero constraints on the random effect terms, we assigned a flat prior on the overall disease risk terms, *α*_*j*_, and the fixed effects were assigned independent Normal(0,10^3^) prior distributions. The logarithms of the scaling parameters were assigned independent Normal(0,5) prior distributions, and the shared component precision parameters *τ*_1_, *τ*_2_, and *τ*_3 _were independently assigned a conjugate hyper-prior Gamma (0.5, 0.0005) distribution[17], which is weakly informative. This implies that, *a priori*, the variances 1/*τ*_1_, 1/*τ*_2 _and 1/*τ*_3 _of the respective shared latent components *φ*_1_, *φ*_2 _and *φ*_3 _follow an inverse gamma distribution with a 99% probability that the variance lies between 0.000151 and 6.25, with a mode at 0.00033, thus concentrated towards 0, which is conservative. This is a reasonable assumption as the differences in risk are likely to be minimal due to relative equity across the region in any information or prevention campaigns in operation and the availability of treatments. In addition, we adjusted for the effects of SEB. The precision matrix Σ^-1 ^for the multivariate normal unstructured random effects was assigned a Wishart(Q,6) prior distribution, where *Q *is set to be a diagonal matrix with 1s. The choice of these hyper-prior distributions of the precision terms may result in some sensitivity of the model results, in particular the overall smoothed relative risks of the cancers.

The model was fitted to the data using full Bayesian estimation within the WinBUGS software [18]. The WinBUGS code used for the shared component model is available as additional file 1 on the IJHG website. For each model, three independent chains were run for 50,000 iterations. We monitored all fixed effects, weight and variance parameters for convergence. We used the Brooks-Gelman-Rubin diagnostic tool [19], which confirmed rapid convergence by 20,000 iterations. Thus, we used the remaining 30,000 iterations from each of the three chains for posterior summaries.

We compared the variations of the joint model using the Deviance Information Criterion (DIC)[20]. The DIC is defined as *DIC *= $\bar{D}$+*p*_*D *_where $\bar{D}$ is the posterior mean of the deviance and measures model fit; and *p*_*D *_is the effective number of model parameters and measures model complexity. The DIC works on similar principles as the non-Bayesian Akaike Information Criterion (AIC). The DIC value of the joint modelling of the six cancers using three shared components was compared to the sum of the DIC values from the six individual BYM models. Summing individual DIC values assumes that the six cancers are independent[16]. The joint model had a DIC value of 18980.5 and the sum of the DIC values from the individual BYM models was 19083.04. This shows a great improvement in the DIC vales for the joint model. The results presented here are from the joint model.

## Results

**Figure 1:**
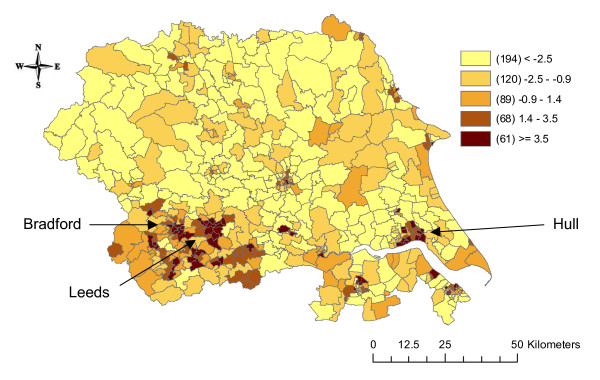
**Map of the 1991 Townsend index of deprivation for each ward in Yorkshire**.

The total population of the Yorkshire region in 1991 was 3,676,305 persons. The minimum number of people living in a ward was 489 and the maximum was 26,705. Figure 1 shows the deprivation score for each ward. The more rural wards in the north of the region were generally more affluent (lighter orange/yellow), whilst the urban areas of Leeds, Bradford, and Hull were more deprived (darker orange/brown). Across the region during the study period there were 7,444 oesophagus cancers, 15,045 stomach cancers, 8,522 pancreas cancers, 54,520 lung cancers, 5,918 kidney cancers, and 15,072 bladder cancers. The mean and range of the ward SIRs for each of the six cancers are shown in Table 1, and the correlations amongst the ward SIRs are shown in Table 2. Cancers of the oesophagus and pancreas were more common than expected in the region, whilst stomach and lung cancer were less common than expected. The correlation amongst the ward SIRs was highest for stomach and lung cancer (0.49), followed by lung and pancreas and lung and bladder (both 0.34). The correlation was lowest between kidney and lung cancer (0.11).

**Table 1:** Mean and range of the standardised incidence ratios for each cancer

	**Mean SIR**	**Minimum SIR**	**Maximum SIR**
**Oesophagus**	1.06	0.00	3.24
**Stomach**	0.94	0.00	2.55
**Pancreas**	1.02	0.00	2.76
**Lung**	0.92	0.07	2.80
**Kidney**	1.00	0.00	2.79
**Bladder**	1.01	0.00	2.58

**Table 2:** Correlations between the standardised incidence ratios for each cancer

	**Oesophagus**	**Stomach**	**Pancreas**	**Lung**	**Kidney**	**Bladder**
**Oesophagus**	1.00	-	-	-	-	-
**Stomach**	0.23	1.00	-	-	-	-
**Pancreas**	0.26	0.23	1.00	-	-	-
**Lung**	0.31	0.49	0.25	1.00	-	-
**Kidney**	0.16	0.15	0.20	0.11	1.00	-
**Bladder**	0.25	0.27	0.34	0.34	0.23	1.00

**Figure 2:**
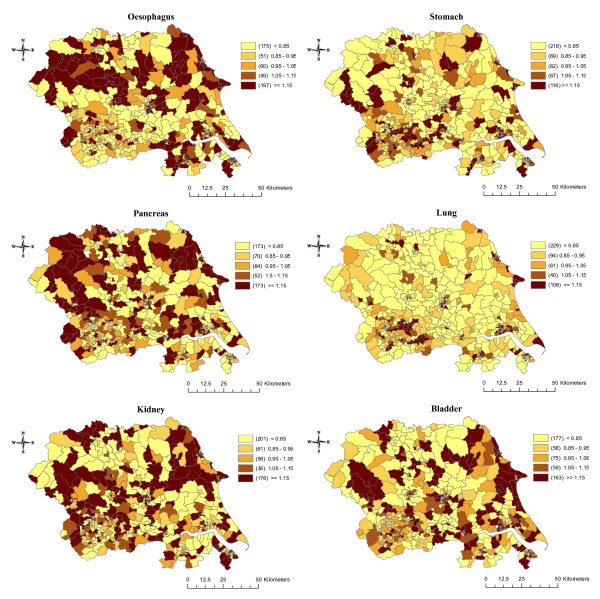
**Maps of the raw (unsmoothed) standardised incidence ratios for six cancers in Yorkshire between 1983 and 2003**.

**Figure 3:**
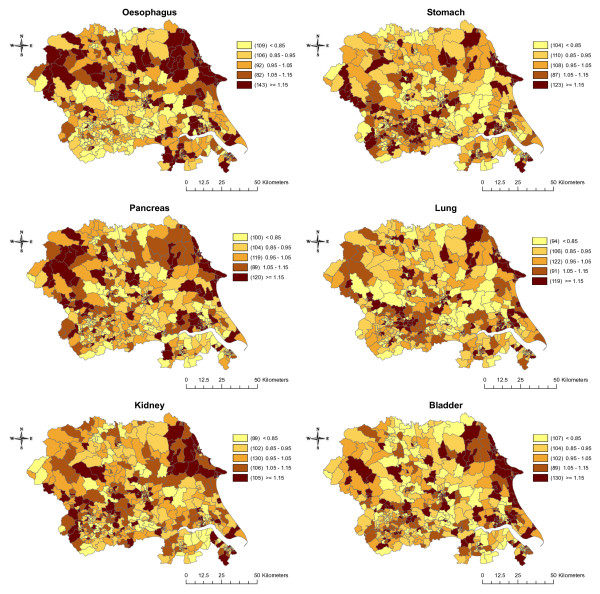
Maps of the posterior median smoothed standardised incidence ratios for six cancers in Yorkshire between 1983 and 2003, adjusted for socioeconomic background.

Figure 2 shows the raw (unsmoothed) SIRs for each cancer. The incidence of cancers of the oesophagus, pancreas, kidney, and bladder appear to be relatively evenly distributed across the region. In contrast, the incidence of stomach and lung cancers were more clustered around the urban areas in the south of the region. As expected, smoothing generally decreased the number of wards with the highest (>= 1.15) and lowest (< 0.85) incidence rates and increased the number of wards in the intermediate categories (results not shown but available on request). The exception to this was an increase in the highest incidence category for lung and stomach cancers. The general patterns seen in the unsmoothed maps remained unchanged. Figure 3 shows the smoothed results after adjustment for SEB. The incidence of lung cancer was most impacted by adjustment for SEB, becoming more evenly distributed across the region, and the concentration of high incidence wards in the urban area of Leeds/Bradford became less apparent. Adjustment for SEB resulted in a similar change for stomach cancer, though to a lesser extent. Adjustment made little difference to the spatial distribution of oesophagus, pancreas, kidney, and bladder cancers.

**Figure 4:**
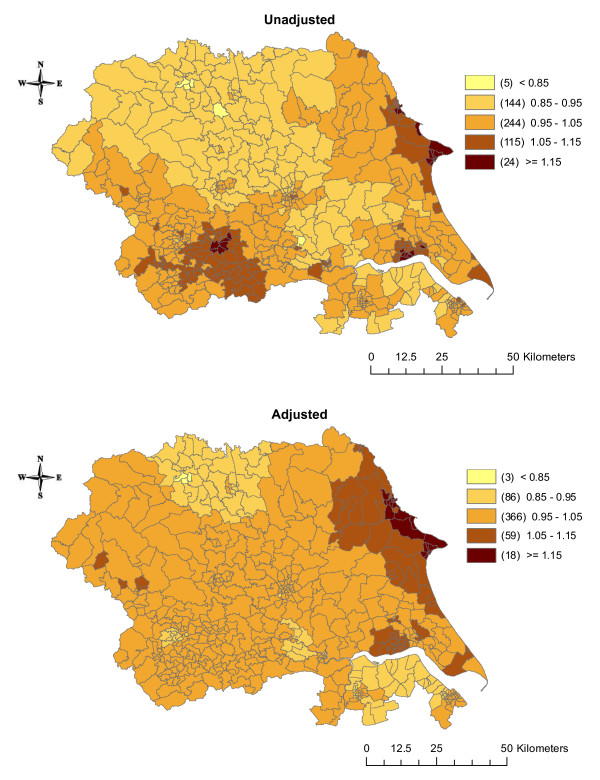
**Maps of the posterior medians of shared component 1 (representing smoking, including all six cancers), unadjusted and adjusted for socioeconomic background**.

**Figure 5:**
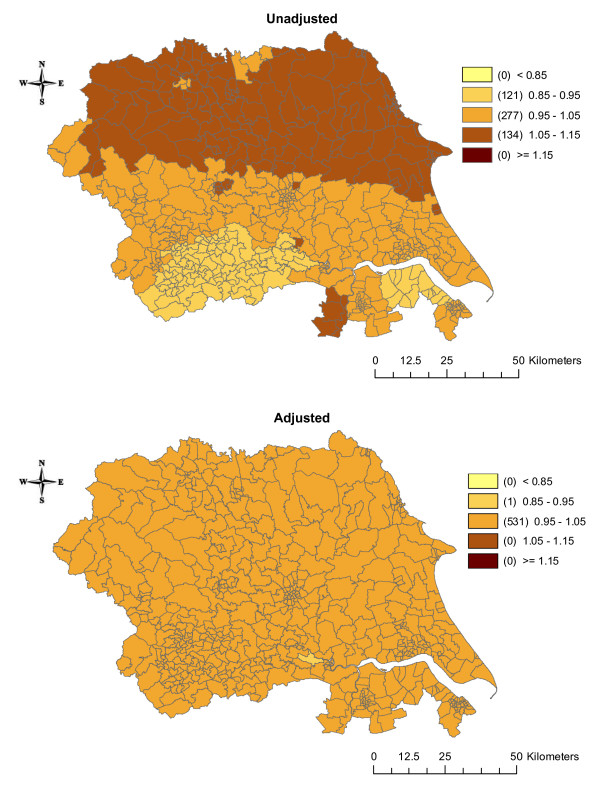
**Maps of the posterior medians of shared component 2 (representing obesity/bodyweight, including oesophagus, pancreas and kidney cancers), unadjusted and adjusted for socioeconomic background**.

**Figure 6:**
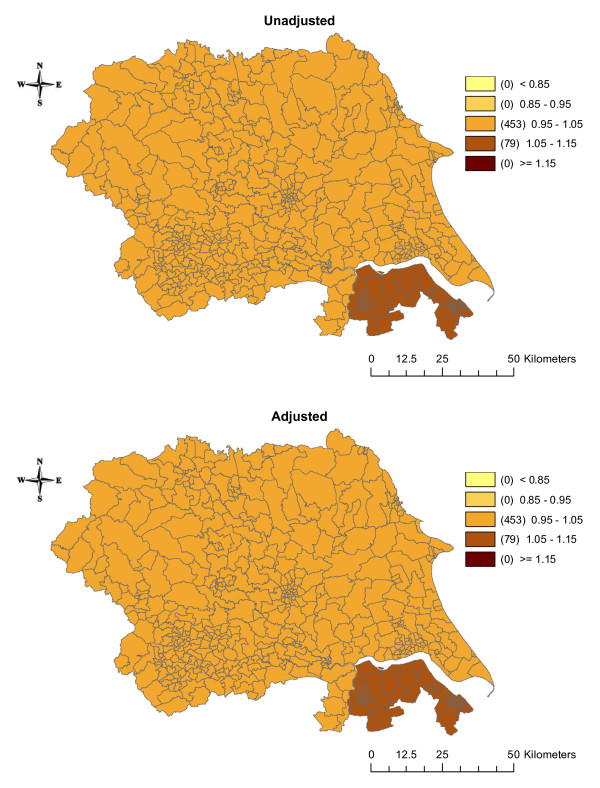
**Maps of the posterior medians of shared component 3 (representing diet/alcohol consumption, including oesophagus and stomach cancers), unadjusted and adjusted for socioeconomic background**.

The estimates of the effects of the three shared components were also mapped. Before adjustment for SEB, component 1 (which was acting as a surrogate for smoking) had a larger effect on cancer incidence in the urban areas around Leeds and in the eastern part of the region (Figure 4). After adjustment, the effect disappeared from around Leeds but became more pronounced in the eastern part of the region. Component 2 (a surrogate for bodyweight/obesity) had a larger effect in the northern part of the region and a smaller effect around Leeds and Bradford in the unadjusted analysis (Figure 5). After adjustment for SEB, these differences disappeared. In the unadjusted analysis, the effect of component 3 (a surrogate for diet/alcohol consumption) showed little variation with an increased effect in Humberside (Figure 6). Adjustment for SEB had no effect on the cancer rates.

Table 3 shows the relative weight, or level of importance, that each shared component has for the different cancers, without adjustment for SEB. Component 1, representing smoking, was more important for lung cancer than for the other five cancers and this was significant relative to all cancers except stomach. In addition, this component was more important for stomach cancer than for the other cancers (except lung cancer) and this was significant relative to oesophagus, pancreas, and kidney cancers. Component 1 was less important for kidney cancer than the other five cancers. This component carried equal weight between stomach and lung cancers, with a ratio of 0.90 (95% CI 0.67–1.17). Shared component 2, representing bodyweight/obesity, was more important for oesophagus cancer relative to pancreas and kidney cancers. This component carried equal weight for pancreas and kidney cancer, with a ratio of 1.05 (95% CI 0.49–2.32). Shared component 3, representing diet/alcohol consumption, was more important for oesophagus cancer relative to stomach cancer, though not significantly so, with a ratio of 2.05 (95% CI 0.56–6.15). Table 4 shows the ratios after adjustment for SEB. In shared component 1, the weight of lung cancer was smaller and the weight of bladder cancer was larger, but none of the results was statistically significant. The results for shared components 2 and 3 showed the same patterns as in the unadjusted analysis.

**Table 3:** Posterior median (95% CI) relative weights of each cancer in the shared components analysis (unadjusted for socioeconomic background)

		**Oesophagus**	**Stomach**	**Pancreas**	**Lung**	**Kidney**	**Bladder**
**Oesophagus**	**1**	1.00					
	**2**	1.00					
	**3**	1.00					
**Stomach**	**1**	0.53 (0.26–0.92)	1.00				
	**2**	-	1.00				
	**3**	2.05 (0.56–6.15)	-				
**Pancreas**	**1**	0.89 (0.46–1.62)	1.67 (1.04–3.02)	1.00			
	**2**	1.72 (0.67–3.59)	-	1.00			
	**3**	-	-	1.00			
**Lung**	**1**	0.48 (0.24–0.80)	0.90 (0.67–1.17)	0.54 (0.30–0.84)	1.00		
	**2**	-	-	-	-		
	**3**	-	-	-	-		
**Kidney**	**1**	1.10 (0.52–2.22)	2.06 (1.19–3.95)	1.23 (0.65–2.42)	2.30 (1.34–4.47)	1.00	
	**2**	1.81 (0.66–4.09)	-	1.05 (0.49–2.32)	-	1.00	
	**3**	-	-	-	-	-	
**Bladder**	**1**	0.81 (0.41–1.45)	1.51 (0.96–2.73)	0.91 (0.52–1.58)	1.69 (1.07–3.05)	0.73 (0.38–1.40)	1.00
	**2**	-	-	-	-	-	-
	**3**	-	-	-	-	-	-

1 – The shared component representing smoking; 2 – The shared component representing bodyweight/obesity; 3 – The shared component representing diet/alcohol consumption.

The figures in the main body of the table represent the weight of the cancer listed along the top row relative to the cancers listed along the left hand side (with 95% confidence intervals). If the RR is > 1.00 the cancer along the top row has more weight, if the RR is < 1.00 the cancer along the left hand side has more weight.

**Table 4:** Posterior median (95% CI) relative weights of each cancer in the shared components analysis (adjusted for socioeconomic background)

		**Oesophagus**	**Stomach**	**Pancreas**	**Lung**	**Kidney**	**Bladder**
**Oesophagus**	**1**	1.00					
	**2**	1.00					
	**3**	1.00					
**Stomach**	**1**	1.06 (0.45–2.20)	1.00				
	**2**	-	1.00				
	**3**	2.29 (0.58–6.47)	1.00				
**Pancreas**	**1**	0.94 (0.47–1.69)	0.89 (0.49–1.68)	1.00			
	**2**	1.89 (0.58–5.16)	-	1.00			
	**3**	-	-	1.00			
**Lung**	**1**	1.09 (0.53–1.95)	1.04 (0.61–1.64)	1.16 (0.65–1.92)	1.00		
	**2**	-	-	-	-		
	**3**	-	-	-	-		
**Kidney**	**1**	1.13 (0.52–2.32)	1.07 (0.57–2.08)	1.20 (0.67–2.28)	1.04 (0.60–1.99)	1.00	
	**2**	2.12 (0.54–5.88)	-	1.10 (0.44–2.61)	-	1.00	
	**3**	-	-	-	-	-	
**Bladder**	**1**	0.75 (0.37–1.29)	0.72 (0.40–1.19)	0.81 (0.47–1.24)	0.69 (0.45–1.02)	0.67 (0.35–1.10)	1.00
	**2**	-	-	-	-	-	-
	**3**	-	-	-	-	-	-

1 – The shared component representing smoking; 2 – The shared component representing bodyweight/obesity; 3 – The shared component representing diet/alcohol consumption.

The figures in the main body of the table represent the weight of the cancer listed along the top row relative to the cancers listed along the left hand side (with 95% confidence intervals). If the RR is > 1.00 the cancer along the top row has more weight, if the RR is < 1.00 the cancer along the left hand side has more weight.

Table 5 shows the relative risk of cancer incidence by SEB fifths. Living in a more deprived area was associated with an increased incidence of lung and stomach cancers (RR = 1.63, 95% CI 1.47–1.78, and RR = 1.33, 95% CI 1.19–1.48, respectively in the most deprived fifth). There also appeared to be a positive association between living in a more deprived area and an increased incidence of oesophagus and pancreas, whilst the opposite was seen for kidney and bladder cancers, although none of these was statistically significant.

**Table 5:** Posterior median (95% CI) relative risk of cancer incidence by Townsend quintile

	**Oesophagus**	**Stomach**	**Pancreas**	**Lung**	**Kidney**	**Bladder**
**I (most affluent)**	1.00	1.00	1.00	1.00	1.00	1.00
**II**	1.11 (0.99–1.24)	1.11 (1.01–1.22)	1.12 (1.01–1.24)	1.12 (1.04–1.20)	1.09 (0.98–1.22)	1.03 (0.94–1.12)
**III**	1.16 (1.03–1.31)	1.24 (1.13–1.37)	1.09 (0.98–1.22)	1.31 (1.21–1.41)	0.96 (0.86–1.09)	1.06 (0.96–1.17)
**IV**	1.16 (1.02–1.32)	1.27 (1.15–1.41)	1.10 (0.98–1.24)	1.47 (1.35–1.59)	0.97 (0.86–1.10)	0.99 (0.89–1.10)
**V (most deprived)**	1.07 (0.93–1.23)	1.33 (1.19–1.48)	1.03 (0.91–1.16)	1.63 (1.47–1.78)	0.89 (0.78–1.01)	0.94 (0.84–1.05)

**Figure 7:**
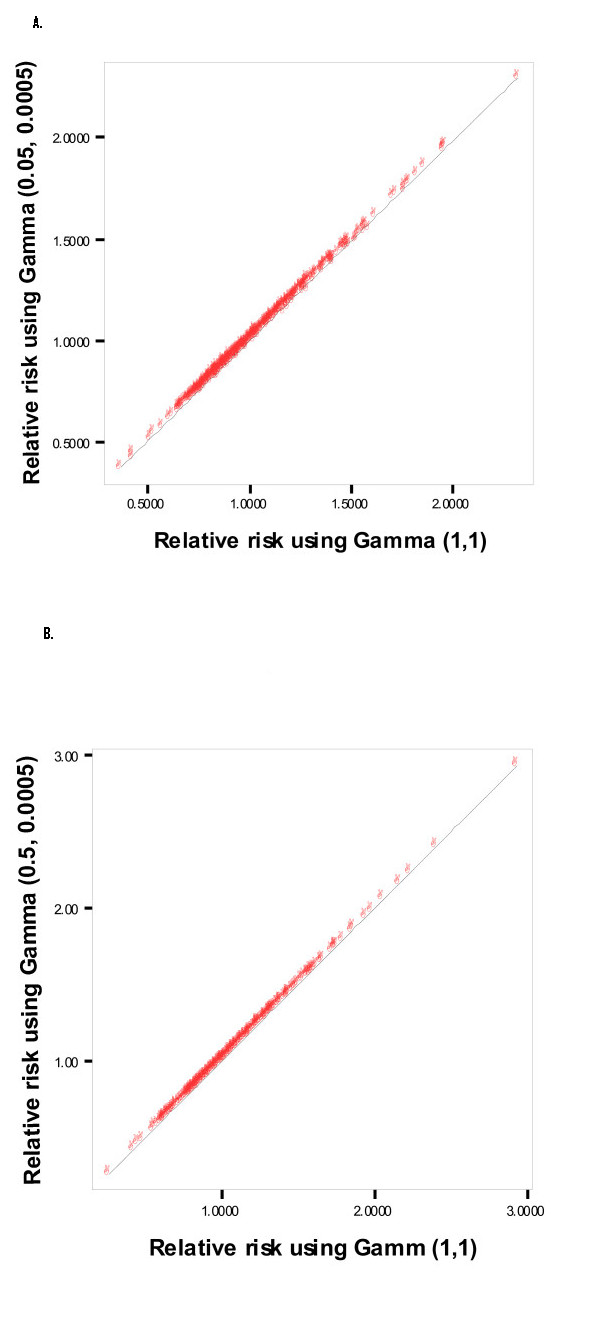
Smoothed relative risks for two precision hyper-prior specifications; stomach (A) and lung cancer (B).

We performed a sensitivity analysis to evaluate variation of the results in response to the choice of the gamma hyper-prior distribution of the precision parameters *τ*_1_, *τ*_2_, and *τ*_3_, we assigned an alternative conjugate hyper-prior Gamma (1,1). Using this specification, the variances 1/*τ*_1_, 1/*τ*_2 _and 1/*τ*_3 _of the respective shared latent components *φ*_1_, *φ*_2_, and *φ*_3 _have a 99% probability of lying between 0.217 and 100, and a mode at 0.5, thus away from 0. We choose to investigate the overall unadjusted smoothed relative risks for lung and stomach cancers, which were most common in the region. Figure 7 shows a comparison of the median smoothed RRs of stomach (A) and lung (B) cancer for the two different specifications of hyper-prior distributions. In both cases, the points lie perfectly on the line of unity, showing agreement of the smoothed risks and confirming previous work showing that the choice of hyper-prior has no effect on the smoothed SIRs[2].

## Discussion

This study demonstrates the feasibility of joint disease modelling using data from six cancer sites. The resulting maps clearly show the geographical differences in the incidence of the six cancers. Smoothing allows us to see the inherent spatial patterns of cancer incidence as the variability or 'noise' has been removed. The estimates are more precise and the associated CIs are smaller than those obtained without smoothing. Generally, the effect of smoothing was to reduce the number of wards in the highest and lowest incidence categories, thereby increasing the number in the intermediate categories. It is well known that after smoothing the RR estimates from areas with small populations are pulled towards the average of 1, while those from areas with larger populations are unchanged from the raw risk[2].

Increasing area deprivation was significantly associated with higher incidence of lung and stomach cancers and there was a clear dose response relationship. Before adjusting for SEB, the incidence of lung and stomach cancers was higher around the urban areas of Leeds and Bradford. Adjusting for SEB weakened this effect, but there was still a cluster of high incidence wards in these areas. The results of the shared components analysis show that the 'smoking' component had more effect in the areas around Leeds, and that the effect was greatest for lung and stomach cancers, but this diminished after adjustment for SEB. Component 2 (representing bodyweight/obesity) was more important in the northern part of the region before adjusting for SEB, but this effect disappeared after adjustment. This component and component 3 (representing diet/alcohol consumption) were relatively more important for oesophagus cancer than the other cancers, but these results were not statistically significant. It may be that the effects of these risk factors are too small to be detected using this methodology. Although the random effects shared components are loosely interpreted to reflect these variables, data on smoking prevalence, levels of obesity and dietary intake are not actually available at such a fine geographical level. Ideally, we would want to include these measures as covariates in the model. In addition, there are other risk factors which could be explored, but their effects are likely to be smaller and not detectable with this type of analysis.

Most cancers have a long latency period, with many years between any exposure to risk factors (e.g. smoking at age 30) and diagnosis of disease (e.g. lung cancer at age 60). Therefore, figures 4, 5, 6 may represent maps of historic smoking prevalence, bodyweight patterns and dietary habits. A possible extension to this work would be to include a temporal component in the model, which may improve the correlations further and could reflect the latency between an exposure and disease, or the effect of a new screening programme or new treatment[21].

In our application we have shown that a joint model of the six cancer sites offers a great improvement over individual BYM models (as demonstrated by the DIC criteria). However, the interpretation of the derived smoothed maps should be treated with caution because, although they are a great improvement over unsmoothed maps, there remain confounding factors, differences in population size, and area factors. In addition, the results may suffer from over- or under-estimation, due to the edge effects phenomenon. We used an adjacency matrix to ascertain the number of neighbours each ward had. However, some wards in the Yorkshire region border wards in other regions. Data were not available for other regions; thus, these wards have missing neighbours, which may introduce some bias when averaging rates or when estimating spatial effects as their variances are inversely related to the total number of areas. Nonetheless, Bayesian hierarchical modelling of relative risks has many advantages over other methods when smoothing across small geographic areas.

This analysis was carried out using data between 1983 and 2003, a period when electoral wards were the most commonly used small geographical unit and populations were available at that level. Super Output Areas (SOAs), introduced in the 2001 census, are now replacing wards and in the near future wards will be obsolete. However, there is no reason why similar studies could not be carried out at the SOA (or other small area) level.

This type of analysis may be useful for authorities to evaluate healthcare system performance and adjust their policies as a result. Such data may also help construct or confirm research hypotheses. In both situations, the aim is to obtain parameter estimates that are as accurate as possible[21]. In this case, we attempted to assess the effect of smoking, bodyweight/obesity, and diet/alcohol on six cancers. It is estimated that over half of all cases of cancer could be prevented through changes to lifestyle, such as quitting smoking, maintaining a healthy weight, and avoiding excessive alcohol consumption. This is therefore an important area of focus and this methodology may prove valuable.

## Competing interests

The authors declare that they have no competing interests.

## Authors' contributions

AD acquired and manipulated the data, carried out the basic statistical analysis and wrote the initial draft of the manuscript. SM conceived the study, carried out the spatial analysis and helped draft the manuscript. KP produced the maps in a GIS package. DF and MG helped with interpretation of the results and revision of the manuscript. All authors read and approved the final version of the manuscript.

## Supplementary Material

Additional file 1 WinBUGS code for shared component model. This file contains the WinBUGS code for the shared component model using the six Yorkshire cancer sites, three shared components and deprivation effects. Click here for file
