# Supplementary material for: Joint disease mapping using six cancers in the Yorkshire region of England
Source: Int J Health Geogr. 2008 Jul 28;7:41. doi: 10.1186/1476-072X-7-41 (PMC2515288; doi:10.1186/1476-072X-7-41)
Supplement: Additional file 1 — WinBUGS code for shared component model. This file contains the WinBUGS code for the shared component model using the six Yorkshire cancer sites, three shared components and deprivation effects. [file 1476-072X-7-41-S1.doc]

**APPENDIX**

WinBUGS code for the shared component model (using the six Yorkshire cancer sites, three shared components and deprivation effects)

Model

{

for (i in 1 : N_wards) {

for (j in 1:Ndiseases) {

# Poisson likelihoods

O[i,j] ~ dpois(mu[i,j])

log(mu[i,j]) <- log(E[i,j])+ alpha[j]+ beta[1,j]*depFifth2[i] + beta[2,j]*depFifth2[i] + beta[3,j]*depFifth2[i] + beta[4,j]*depFifth2[i] + epsilon[i,j] +v[i,j]

eta[i,j] <- u[i,j] +v[i,j]

}

}

for(i in 1:N_wards) {

# Define log relative risk in terms of disease-specific (psi) and shared (phi) random effects, with weights (delta)

v[i,1] <- delta1[1]*phi1[i] + delta2[1]*phi2[i] + delta3[1]*phi3[i]

v[i,2] <- delta1[2]*phi1[i] + delta3[2]*phi3[i]

v[i,3] <- delta1[3]*phi1[i] + delta2[2]*phi2[i]

v[i,4] <- delta1[4]*phi1[i]

v[i,5] <- delta1[5]*phi1[i] + delta2[3]*phi2[i]

v[i,6] <- delta1[6]*phi1[i] }

# Spatial prior model for the shared random effects

phi1[1:N_wards] ~ car.normal(adj[], weights[], num[], tau.spatial[1])

phi2[1:N_wards] ~ car.normal(adj[], weights[], num[], tau.spatial[2])

phi3[1:N_wards] ~ car.normal(adj[], weights[], num[], tau.spatial[3])

# Weights for the spatial adjacency matrix (2902 is total numbers of neighbours)

for (k in 1:2902) {weights[k] <- 1 }

# Multivariate Normal distribution for the unstructured components

for (i in 1:N_wards) {

epsilon[i,1:6] ~ dmnorm(mean[1:6], P[1:6, 1:6])

}

# Priors for the intercepts, fixed effects, relative weight and variances

for (j in 1:Ndiseases) {

alpha[j] ~ dflat()

RR.alpha[j]<-exp(alpha[j])

}

for (j in 1:shared1) {

logdelta1[j] ~ dnorm(0, 5.9)

delta1[j] <- exp(logdelta1[j])

}

for (j in 1:shared2) {

logdelta2[j] ~ dnorm(0, 5.9)

delta2[j] <-exp(logdelta2[j])

}

for (j in 1:shared3) {

logdelta3[j] ~ dnorm(0, 5.9)

delta3[j] <-exp(logdelta3[j])

}

for (k in 1:N_shared) {

tau.spatial[k] ~ dgamma(0.5, 0.0005)

sigma2.spatial[k] <-1/tau.spatial[k]

}

for (l in 1:lbeta) { for (k in 1:Ndiseases) {beta[l,k] ~ dnorm(0,0.001)}}

for (l in 1:lbeta) { for (k in 1:Ndiseases) {RRb[l,k] <-exp(beta[l,k]) }}

for (j in 1:6) {mean[j] <-0.0 }

P[1:6,1:6] ~ dwish(Q[1:6, 1:6],6)

}
